# 1704. *Stenotrophomonas maltophilia* Infection in a Neonatal Intensive Care Unit (NICU): A Retrospective Study of Risk Factors and Outcomes in an Inner-City Hospital

**DOI:** 10.1093/ofid/ofad500.1537

**Published:** 2023-11-27

**Authors:** Susannah D Franco, Margaret Hammerschlag, Ashraf Abdelhemid, Lawrence Fordjour, Stephan Kohlhoff

**Affiliations:** SUNY Downstate Medical Center, Brooklyn, New York; SUNY Downstate Medical Center, Brooklyn, New York; SUNY Downstate Medical Center, Brooklyn, New York; SUNY Downstate Medical Center, Brooklyn, New York; SUNY Downstate Medical Center, Brooklyn, New York

## Abstract

**Background:**

*Stenotrophomonas maltophilia* is an aerobic, non-fermenting, Gram-negative bacillus associated with opportunistic and multi-drug resistant infections. The objective of this study was to identify risk factors and outcomes associated with *S. maltophilia* isolation or infection in a level IIIB neonatal intensive care unit (NICU).

**Methods:**

This was a retrospective matched case-control study. Cases were matched 2:1 for birth weight, gestational age, and year of NICU admission. The Chi-square test and Fisher’s exact test were used for comparisons of categorical variables. Continuous variables were analyzed with the Mann-Whitney U test.

**Results:**

A total of 15 *S. maltophilia* isolates were identified from 15 neonates between January 1, 2008 and April 1, 2020 and matched to 30 controls. Median birth weight and gestational age of the cases was 675 g (IQR, 522.5-997.5 g) and 26 weeks (IQR, 24.86-27.14 weeks), respectively. The trachea was the most frequent site of isolation (9 of 15 isolates); colonization was suspected in 2 cases. Other sites included blood (1 case), conjunctiva (2 cases), and peritoneum (3 cases). The following factors were associated with risk of isolation or infection in cases compared to controls: total days of antibiotic exposure (24 vs 15 days, *P* = 0.01), total number of antibiotic courses (3 vs 2.5, *P* = 0.043), total days of broad-spectrum antibiotic exposure (19 vs 9.25 days, *P* = 0.015), and total days of meropenem exposure (9 vs 3 days, *P* = 0.008). Total length of NICU stay was significantly longer among cases versus controls (134 vs 68.5 days, *P* = 0.006). Mortality rate was also significantly higher in cases versus controls (N = 15, 33.33% vs N = 30, 0%; *P* < 0.001); 3 of 3 infants with peritoneal infection died. All 15 isolates were initially susceptible to levofloxacin, and 14 were susceptible to trimethoprim-sulfamethoxazole.

**Conclusion:**

Broad-spectrum antibiotic exposure, especially to meropenem, may lead to selection of resistant bacteria and promote *S. maltophilia* colonization and infection. *S. maltophilia* isolation or infection was associated with increased length of NICU stay and mortality.

**Disclosures:**

**All Authors**: No reported disclosures

